# A Microwave Method for Dielectric Characterization Measurement of Small Liquids Using a Metamaterial-Based Sensor

**DOI:** 10.3390/s18051438

**Published:** 2018-05-05

**Authors:** Weina Liu, Haoran Sun, Lei Xu

**Affiliations:** 1 College of Electric and Electrical Engineering, Henan Normal University, Xinxiang 453007, China; 2013070@htu.edu.cn; 2 College of Electronic Engineering, Chengdu University of Information Technology, Chengdu 610225, China; sunhaoran@cuit.edu.cn; 3 School of Information Engineering, Southwest University of Science and Technology, Mianyang 621010, China

**Keywords:** complex permittivity, microwave measurement, split-ring resonator, sensor, sub-wavelength

## Abstract

We present a microwave method for the dielectric characterization of small liquids based on a metamaterial-based sensor The proposed sensor consists of a micro-strip line and a double split-ring resonator (SRR). A large electric field is observed on the two splits of the double SRRs at the resonance frequency (1.9 GHz). The dielectric property data of the samples under test (SUTs) were obtained with two measurements. One is with the sensor loaded with the reference liquid (REF) and the other is with the sensor loaded with the SUTs. Additionally, the principle of extracting permittivity from measured changes of resonance characteristics changes of the sensor loaded with REF and SUTs is given. Some measurements were carried out at 1.9 GHz, and the calculated results of methanol–water mixtures with different molar fractions agree well with the time-domain reflectometry method. Moreover, the proposed sensor is compact and highly sensitive for use of sub-wavelength resonance. In comparison with literature data, relative errors are less than 3% for the real parts and 2% for the imaginary parts of complex permittivity.

## 1. Introduction

Generally, materials can be divided into electric and magnetic media. Dielectric materials are represented only using relative complex dielectric constant, while magnetic materials are denoted by relative complex dielectric constant and magnetic conductivity. Therefore, for dielectric materials, complex permittivity measurement plays an important role in microwave engineering, and is essential study in a number of applications [1,2,3], such as biological materials and cells [4], mixtures of coal and limestone, and electromagnetic compatibility (EMC) [5,6,7,8].

Dielectric spectroscopy that measures dielectric properties operates in real-time, is on-line 24 h a day, is label-free, is easy to integrate with other microwave circuit, and yields high-volume production [9]. Therefore, there are different methods of the complex permittivity determination of dielectric materials, including transmission-only, reflection-only, transmission-reflection, and resonant cavity methods [10,11,12,13,14,15,16,17,18]. These methods can be divided into two categories: resonant and non-resonant. In all methods, resonance technology has the highest accuracy and is used to test low-loss materials. In the classical resonance method, the SUT is placed into a resonant cavity, and the electromagnetic characteristics of the SUT can then be estimated from the change of the quality factor (Q) and resonant frequency of the cavity. However, this method suffers from a narrow band, complicated calibration procedures, and a destructed test for dielectric materials.

Recently, a new type of microwave resonance device has been developed using artificial electromagnetic materials (meta-materials) [19,20,21]. Meta-material-inspired devices are employed to ensure dielectric characterization of the SUT because of their compactness and high Q-factor. Furthermore, they are made of sub-wavelength resonators and are extremely sensitive to environment changes. Several novel or modified microwave and terahertz sensors using meta-materials are being presented for various measurement applications, for instance, thin-film and electrically small sample sensing, rotation, displacement, and strain sensing [22,23,24].

In the paper, we propose and demonstrate a new two-port metamaterial-based microwave sensor to obtain a non-invasive measurement of complex permittivity of small liquids. The complex permittivity is determined from changes in the magnitude and phase of the transmission coefficient of the sensor loaded and unloaded with the SUT. Moreover, the sensor is designed to work at 1.9 GHz and can be fabricated using PCB technology. Therefore, it satisfies the demands for miniaturization, a compact size, a low cost, and high sensitivity in sensing applications.

## 2. Theoretical Analysis

**Figure 1:**
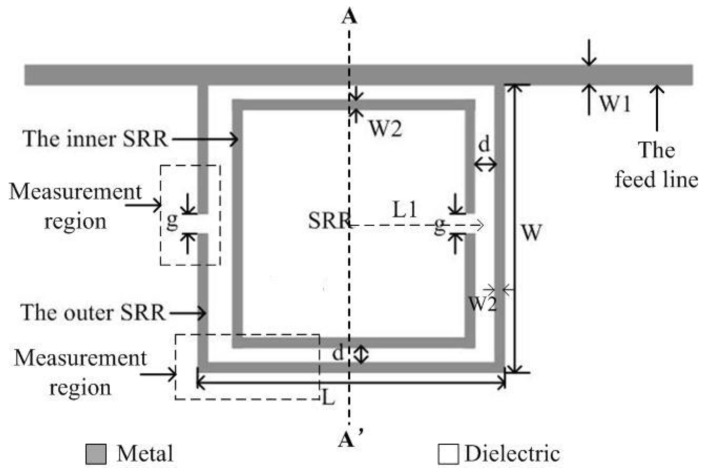
Layout of the proposed sensor with dimensions as follows: *W*1 = 1.7 mm, *g* = 0.9 mm, *d* = 1 mm, *W*2 = 0.9 mm, *W* = 10 mm, *L* = 10 mm, and *L*1 = 3.1 mm.

As sketched in Figure 1, the proposed sensor consists of a micro-strip line and two SRRs. Each SRR is composed of a square metal ring with a split upon one side. Two splits are symmetrical about the plane AA’ and the middle section of the micro-strip line is overlapped one side of the outer SRR as shown in Figure 1. The metallic structure is also supported by the ground plane on its bottom side. By doing so, the dimension of the SRR can be reduced to less than one wavelength to increase the resonant Q factor. Moreover, multiple solutions can be avoided for two-port measurement. Since the SRRs are etched in the same plane with the micro-strip line, which is used to excite the electric field of the SRRs. The complex permittivity of samples is determined from the measured information of the sensor, which is suitable to analyze the concentration and determine the composition of liquids [21]. The proposed method is contact and nondestructive for the SUTs directly placed on the region with the strongest electrical field.

**Figure 2:**
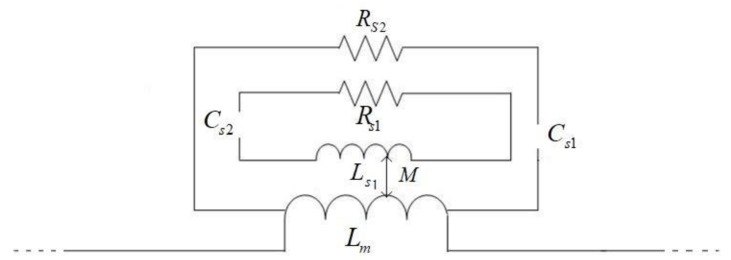
Equivalent circuit of the proposed sensor.

From [19], we know that SRRs behave as an LC resonator, which can be excited by an external magnetic flux, exhibiting a strong diamagnetism above their first resonance. SRRs also exhibit cross-polarization effects (magneto-electric coupling) so that excitation by a properly polarized time-varying external electric field is also possible. Therefore, the equivalent-circuit mode of the sensor can be expressed as Figure 2. An incoming microwave signal a1 from Port 1 will arrive to Port 2 via the SRRs. The quasi-TEM electromagnetic wave propagation mode developed in the micro-strip line will excite the SRRs by the side overlapped the feed-line. Specifically, the SRR ring and gap are accountable for the electrical inductance $L_{s}$ and the capacitance $C_{s}$ respectively, as shown in Figure 2. Therefore, the SRRs form a series RLC resonant circuit and the impedance of the resonance device can be expressed as

(1) $$Z_{s}=R_{s}+j\omega L_{s}+\frac{1}{j\omega C_{s}}.$$

If we do not take into account the mutual inductance M between the SRR and feed line. Then,

(2) $$C_{s}=\frac{C_{s1}C_{s2}}{C_{s1}+C_{s2}},\;L_{s}=\frac{L_{s}L_{m}}{L_{s1}+L_{m}},\;R_{s}=\frac{R_{s1}R_{s2}}{R_{s1}+R_{s2}}.$$

The resonance frequency and Q factor are

(3) $$f_{0}=\frac{1}{2\pi \sqrt{L_{S}C_{S}}},\;Q=R_{S}\sqrt{\frac{C_{S}}{L_{S}}}.$$

If the SUT is placed on the measurement region of the sensor, the capacitor $C_{s}$ is affected by changes of the dielectric property of it. Therefore,

(4) $$C_{S}=C_{0}+\varepsilon_{SUT}C_{e}$$

where *C*_0_ represents the parasitic capacitance. $C_{e}$ is the capacitance of the measurement region of the empty sensor. $\varepsilon_{SUT}$ denotes the complex permittivity of the SUT and can be defined as:

(5) $$\varepsilon_{SUT}=\varepsilon_{SUT}^{\prime }+j\varepsilon_{SUT}^{\prime \prime }.$$

Thus, from Equations (1)–(4), we can obtain the relation expression for complex permittivity of the SUT, which is the function of the resonance frequency and the Q factor of the sensor and can be expressed as

(6) $$f_{0}=\frac{1}{2\pi \sqrt{L_{S}\left[C_{0}+(\varepsilon_{SUT}^{\prime }+j\varepsilon_{SUT}^{\prime \prime }C_{e})\right]}}=F_{x}(\varepsilon_{SUT}^{\prime },\varepsilon_{SUT}^{\prime \prime })\;Q=R_{S}\sqrt{\frac{\left[C_{0}+(\varepsilon_{SUT}^{\prime }+j\varepsilon_{SUT}^{\prime \prime }C_{e})\right]}{L_{S}}}=F_{y}(\varepsilon_{SUT}^{\prime },\varepsilon_{SUT}^{\prime \prime }).$$

## 3. Simulations and Experiments

**Figure 3:**
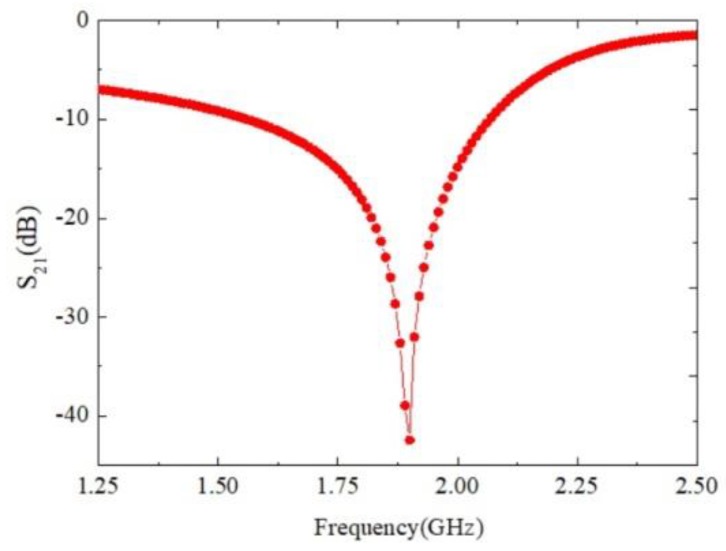
Simulated resonance of the proposed sensor.

In order to validate the proposed method, a prototype described in Section 2, was designed on a Rogers R4350B substrate with relative dielectric constant $\varepsilon_{r}=3.48$, substrate thickness h=0.76 mm, and top copper foil thickness t=18 μm. The performance of the sensor was simulated with ANSOFT HFSS in the bandwidth from 0.8 to 3.5 GHz. The simulated results of the empty sensor are shown in Figure 3. It can be seen that the transmission magnitude is lower than −42 dB at the resonant frequency 1.9 GHz, namely, a very high-Q factor resonance of the SRRs.

As sketched in Figure 1 and Figure 2, $C_{s1}=C_{s2}=\frac{PC_{pul}}{2}$, where *P* is the perimeter of the square ring with the side length 2L1, and $C_{pul}$ is the per unit length capacitance between the rings, while the inductance $L_{s}$ can be approximated by that of a single ring with average perimeter Side *L1* and width C. Moreover, $C_{pul}$ can be obtained from the well-known expression: $C_{pul}=\frac{\beta }{\omega Z_{0}}$. β and $Z_{0}$ denote the propagation constant and characteristic impedance of a micro-strip line. Therefore, the inferred element values of the equivalent circuit model are $C_{s}=0.99\;\mathrm{pF},L_{s}=1.19\;\mathrm{nH}$.

**Figure 4:**
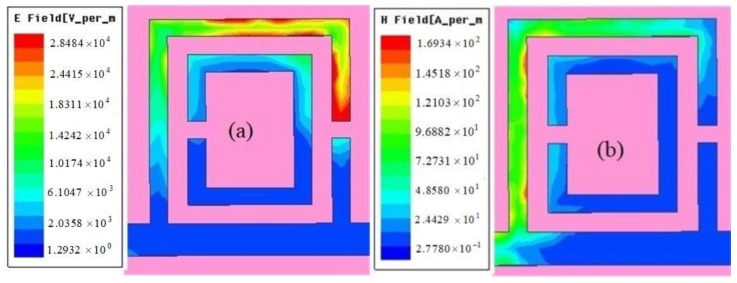
Simulation electric field (**a**) and magnetic field (**b**) distributions on the top side of the sensor.

Furthermore, a large electric field is observed on the two splits of the double SRRs at 1.9 GHz. The strongest electric field is distributed in the two regions of the outer SRR as shown in Figure 4a: one is the split of the outer SRR, and the other is in parallel to the feed line. Therefore, the SUT is placed on the above-mentioned two areas, namely the measurement region, which can ensure an electric field that can affect the SUT and improve the measurement sensitivity. We also present the magnetic field distribution on the SRR of the sensor. It can be observed that a large magnetic field is located in the side of the outer SRR that is nearest to the split of the inner SRR, whereas, in the strongest part of the electric field, that is, at the split of the outer SRR, the magnetic field is very weak.

**Figure 5:**
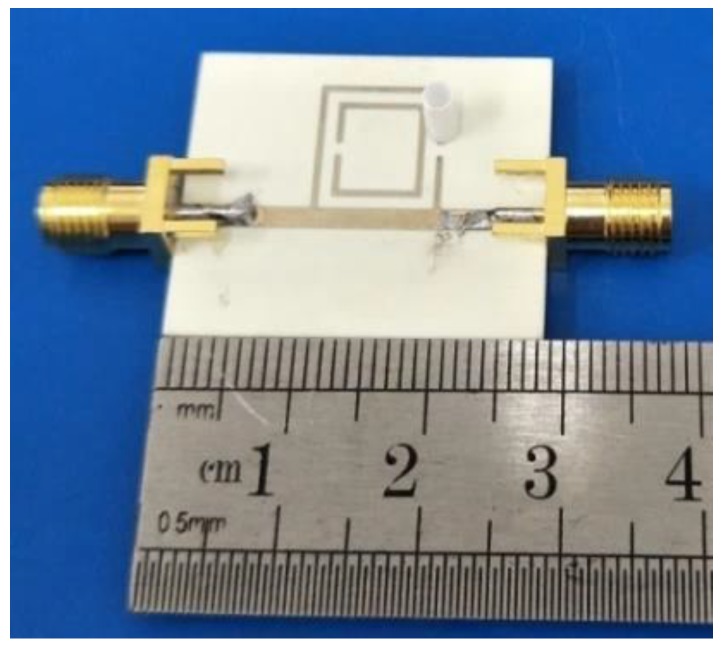
The fabricated sensor with a plastic pipe attached.

The SRR-based sensor was manufactured and is displayed in Figure 5. The substrate is also the Rogers R4350B high-frequency laminate with a thickness of 0.762 mm, a relative permittivity of 3.48, a loss tangent of 0.0027, and a gold-coated copper foil thickness of 0.017 mm.

In order to inject sample solution, a plastic pipe whose diameter and height equal 2 mm and 8 mm, respectively, was glued by the epoxy silicone adhesive on the measurement region. A photograph of the fabricated sensor with the proposed plastic pipe attached is shown in Figure 5. Some experiments are carried out using a vector network analyzer (AV3629D) and the fabricated sensor.

**Figure 6:**
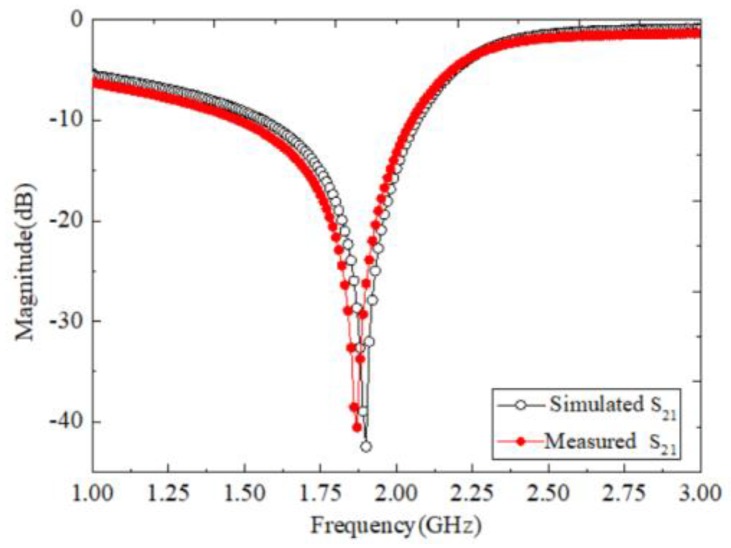
Simulated and measured *S*_21_ of the sensor in the unload case.

In Figure 5, Ports 1 and 2 were jointed with a vector network analyzer via the same two SMA connectors, whose characteristic impedances are 50 Ω. The simulated and measured transmission response of the sensor unloaded with the SUT is displayed in Figure 6. We found that the measured result agrees well with that of the simulation. The difference between them can be attributed to the measurement system, the environment, and the attached plastic pipe.

## 4. Sensor Characteristics

From Equations (1)–(6), a simple model between the complex permittivity and the resonance characteristics of the sensor is obtained as follows:

(7) $$\Delta f_{0}=m_{1}\Delta \varepsilon_{SUT}^{\prime }+m_{2}\Delta \varepsilon_{SUT}^{\prime \prime }$$

(8) $$\Delta \left|S_{21}\right|=n_{1}\Delta \varepsilon_{SUT}^{\prime }+n_{2}\Delta \varepsilon_{SUT}^{\prime \prime }$$

where $\Delta \varepsilon_{SUT}^{\prime }=\varepsilon_{SUT}^{\prime }-\;\varepsilon_{REF}^{\prime },\Delta \varepsilon_{SUT}^{\prime \prime }=\varepsilon_{SUT}^{\prime \prime }-\varepsilon_{REF}^{\prime \prime },$ and $\Delta f_{0}=f_{0}{}_{SUT}-f_{0}{}_{REF}$. The subscript SUT and REF denote the samples under test and the reference sample, respectively. In this paper, the de-ionized water is considered as the reference. $f_{0}{}_{SUT}$ and $f_{0}{}_{REF}$ are the resonant frequency of the sensor each loaded with the SUTs and the reference sample. Furthermore, $\varepsilon_{ref}^{}=\varepsilon_{ref}^{\prime }+j\varepsilon_{ref}^{\prime \prime }$. The harmonic frequency $f_{ref}$ and quality factor *Q_ref_* are generated by the reference sample.

The $m_{1},m_{2},n_{1},n_{2}$ in Equations (7) and (8) are undetermined coefficients. They can be determined from the measured resonance parameters of two samples with known complex permittivity.

**Figure 7:**
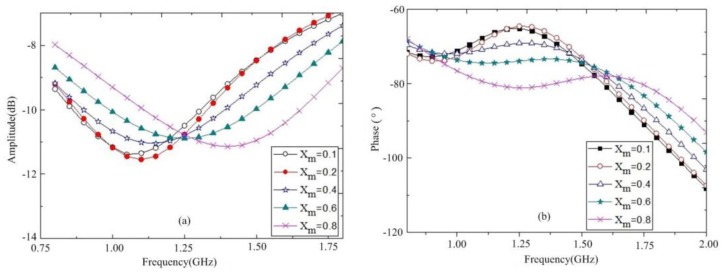
Measured transmission responses for the mixtures of methanol and de-ionized water. (**a**) Amplitudes; (**b**) phases.

Some experiments were carried out using the proposed sensor and a vector network analyzer (AV3629D). Methanol–water mixtures with different molar fractions were used to determine the coefficients of $m_{1},m_{2},n_{1},$ and $n_{2}$, whose complex permittivity has been well studied in the microwave regime [25]. In this measurement, different molar fractions of methanol ($X_{m}$) in the methanol–water mixtures were changed, from $X_{m}=0.1,\;0.2,\;0.4,\;0.6$ to $X_{m}=0.8$. Moreover, every resonant frequency and attenuation of the sensor loaded with different samples was recorded. The results are shown in Figure 7.

As seen, the resonant frequency shifts from 1.9 down to 1.15 GHz, corresponding to the methanol molar fraction increases from $X_{m}=0.1,\;0.2,\;0.4,\;0.6$ to $X_{m}=0.8$. The peak attenuation also changes with different measured samples, and the minimum and maximal peak attenuations are $X_{m}=0.1$ and $X_{m}=0.8$, respectively. Remarkably, the peak attenuation of the sensor with different samples changes nonlinearly. It might be that, between the water content and loss, there is a nonlinear function for water mixtures. According to the Debye model equation, the complex permittivity of the methanol–water mixtures can be denoted as

(9) $$\varepsilon_{SUT}=\varepsilon_{\infty }+\frac{\Delta \varepsilon_{D}}{1-j\omega \tau_{D}}.$$

**Figure 8:**
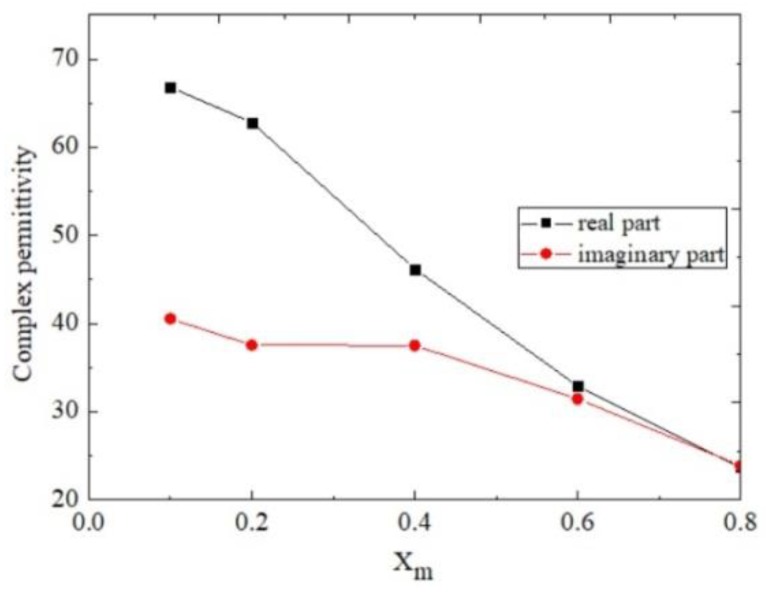
Complex permittivity of methanol–water mixture at 1.9 GHz. The values are taken from [25].

The high frequency relative permittivity $\varepsilon_{\infty }$, dielectric decrement $\Delta \varepsilon_{D}$, and Debye relaxation time $\tau_{D}$ are obtained from the literature [25]. The calculated complex permittivity of the different molar fraction methanol–water mixtures at 1.9 GHz is displayed in Figure 8.

Based on these datasets, the coefficients of $m_{1},m_{2},n_{1},$ and $n_{2}$ shown in Equations (7) and (8) are obtained, and the matrix expression between the complex permittivity and the resonance characteristics are yielded as follows:

(10) $$\left[\begin{array}{l} \Delta f_{0}\\ \Delta \left|S_{21}\right| \end{array}\right]=\left[\begin{array}{ll} 0.0000686 & 0.007154\\ 7.984 & -0.019 \end{array}\right]\left[\begin{array}{l} \Delta \varepsilon_{SUT}^{\prime }\\ \Delta \varepsilon_{SUT}^{\prime \prime } \end{array}\right].$$

By comparing Equations (7), (8), and (10), it can be observed that $\left|m_{2}\right|\approx 100\left|m_{2}\right|$, $\left|n_{1}\right|\approx 400\left|n_{2}\right|$. Therefore, Equation (9) is simplified as

(11) $$\left[\begin{array}{l} \Delta f_{0}\\ \Delta \left|S_{21}\right| \end{array}\right]=\left[\begin{array}{cc} 0 & 0.007154\\ 7.984 & 0 \end{array}\right]\left[\begin{array}{l} \Delta \varepsilon_{SUT}^{\prime }\\ \Delta \varepsilon_{SUT}^{\prime \prime } \end{array}\right].$$

## 5. Results and Discussions

**Figure 9:**
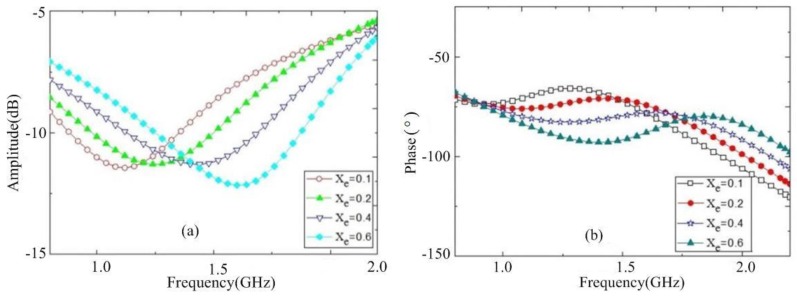
Measured transmission responses for the mixtures of ethanol and de-ionized water. (**a**) Amplitudes; (**b**) phases.

To verify the proposed sensor model in Equation (11), binary mixtures of ethanol and de-ionized water were employed. In this measurement, the molar fraction of methanol ($X_{e}$) was changed from $X_{e}=0.1,0.2,0.4$ to $X_{e}=0.6$. Figure 9 shows the measured responses of transmission parameters (including resonance frequency, peak attenuation, and phase) of the sensor with these liquids. It can be observed that the corresponding frequency shift for $X_{e}=0.1$ to $X_{e}=0.6$ of the ethanol molar fraction is about 500 MHz, which is five times more sensitive with respect to results from [21] showing a 100 MHz shift.

**Figure 10:**
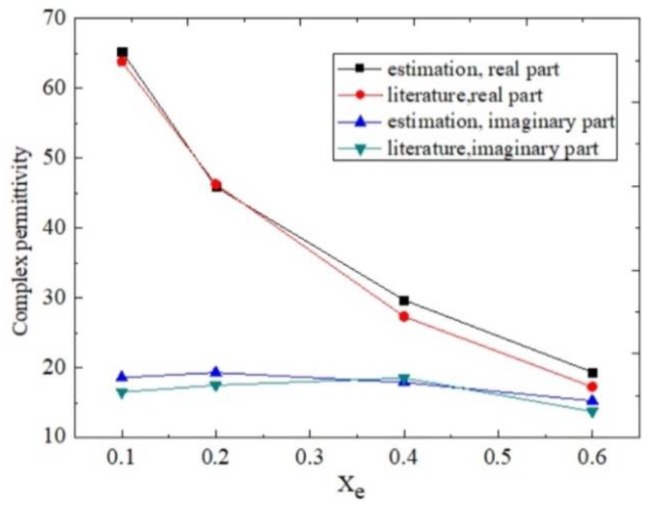
Complex permittivity of methanol–water mixtures. The literature values are taken from [25].

During testing, the reference sample (REF) is the mixture of methanol and de-ionized water with $X_{m}=0.4$. Then, the sample complex permittivity can be determined from Equation (11) using the measured changes of resonance characteristics of the sensor loaded with different SUTs. The calculated results are shown in Figure 10.

We can observe that an acceptable accuracy of the proposed method is demonstrated. The small disagreements of complex permittivity between the measurements and those of [25] are mainly due to the test uncertainties and approximation of the equation [11]. The proposed method can also be employed to measure other liquids covering a wider range of dielectric constant values to establish a much more precise calculating model.

The obtained results from the proposed method have been demonstrated by the comparison with other published literature values of the same SUTs. There are two main advantages of the presented sensor. One is that the sensor has the ability to avoid multiple solutions of extracting dielectric properties of the SUTs for the two-port test, and the other is that the measurement sensitivity is greatly increased because of the overlap of the middle section of the feeder line and one side of the outer SRR.

**Figure 11:**
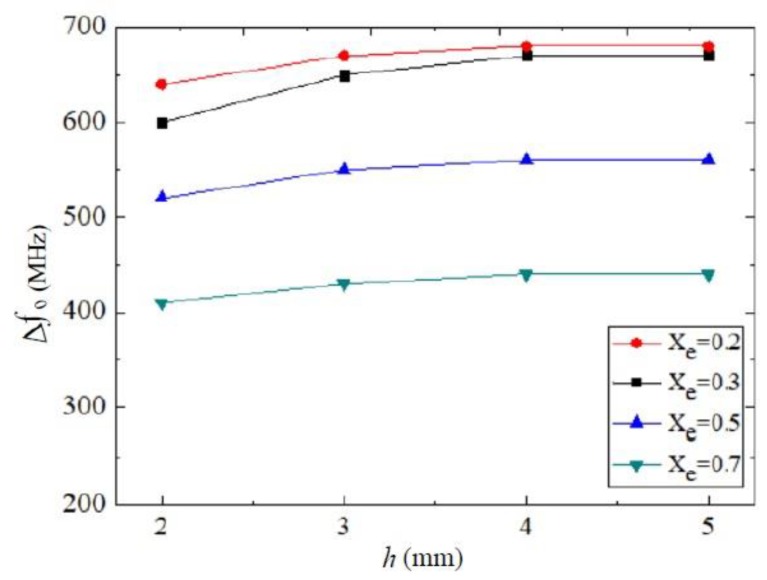
Variation of $f_{0}^{}$ with ethanol–water mixture at different height h. The radius of the test pipe is always 3 mm in the experiments and $X_{e}$ is the molar fraction of ethanol–water mixture.

To illustrate the factor that influences the accuracy of the device. Figure 11 shows the measured variation of frequency $f_{0}$ with different ethanol–water mixtures at a different height *h* of the test plastic pipe.

During the experiment, the test plastic pipes have the same diameter (2 mm). The higher the height *h* is, the larger the frequency shifts are for the same SUT. Therefore, the test accuracy of the sensor is affected by the volume of SUT. However, the height of the SUT will not influence the test result of the device if *h* is greater than or equal to 4 mm, as can be seen from Figure 11, because two curves (*h* = 4 mm and *h* = 5 mm) are already parallel. That is, only a small amount of sample volume (50.3 mm^3^) is required for the sensor to generate the necessary frequency variations.

Compared with the methods found in [26,27,28,29], the proposed method has the following two advantages: one is that the one side of the sensor overlaps the middle section of the feed line. This king of incentive method ensures that the electric field strength of the measurement region achieves $2.8\;\times \;10^{4}\;V/m$. Therefore, on resonance, the magnitude of the transmission coefficient is lower than −42 dB. That is, we obtain a very high sensitive sensor. The other is the simplified calculation equation about the complex permittivity of the SUT is proposed. Furthermore, we identify the factor influencing the accuracy of the sensor. That is, only a 4 mm height of the SUT is required for the device to obtain accurate measurement results when the diameter of the test plastic pipe is 2 mm.

## 6. Conclusions

A simple and meta-material-based microwave sensor for measurement complex permittivity of small liquid is presented. The sensor has been designed by the micro-strip line technology. It includes double SRRs—namely, the outer and inner SRR, and a micro-strip line. Specially, the middle part of the feed line and one side of the outer SRR overlap. Therefore, the sensitivity of the sensor is dramatically improved. Additionally, the complex permittivity of the SUTs can be obtained by two simple measurements. One is that the sensor loaded with the reference liquid and the other is the sensor loaded with the SUTs. Furthermore, we provide a simplified equation to extract complex permittivity from the measured changes of resonance characteristics of the sensor. By using sub-wavelength resonance, the sensor has a very compact size and can be integrated with other microwave circuits.

The experiments with liquid binary mixtures, including ethanol–water and methanol–water, successfully validate the concept. Owing to its capability of composition quantification and characterization, the proposed sensor and its associated technique are promising for dynamic environments in analytical chemistry. Further work will consist in developing a data processing method and the design of an SRR-inspired sensor with multiple ports. We shall also conduct broadband measurement technology using other metamaterial-based sensors.
